# Computational imaging with a balanced detector

**DOI:** 10.1038/srep29181

**Published:** 2016-06-29

**Authors:** F. Soldevila, P. Clemente, E. Tajahuerce, N. Uribe-Patarroyo, P. Andrés, J. Lancis

**Affiliations:** 1 GROC·UJI, Institute of New Imaging Technologies (INIT), Universitat Jaume I, E12071 Castelló, Spain; 2 Servei Central d’Instrumentació Científica (SCIC), Universitat Jaume I, E12071 Castelló, Spain; 3 Wellman Center for Photomedicine, Harvard Medical School and Massachusetts General Hospital, 40 Blossom Street, Boston, Massachusetts 02114, USA; 4 Facultat de Física, Universitat de València, E46100 Burjassot, Spain

## Abstract

Single-pixel cameras allow to obtain images in a wide range of challenging scenarios, including broad regions of the electromagnetic spectrum and through scattering media. However, there still exist several drawbacks that single-pixel architectures must address, such as acquisition speed and imaging in the presence of ambient light. In this work we introduce balanced detection in combination with simultaneous complementary illumination in a single-pixel camera. This approach enables to acquire information even when the power of the parasite signal is higher than the signal itself. Furthermore, this novel detection scheme increases both the frame rate and the signal-to-noise ratio of the system. By means of a fast digital micromirror device together with a low numerical aperture collecting system, we are able to produce a live-feed video with a resolution of 64 × 64 pixels at 5 Hz. With advanced undersampling techniques, such as compressive sensing, we can acquire information at rates of 25 Hz. By using this strategy, we foresee real-time biological imaging with large area detectors in conditions where array sensors are unable to operate properly, such as infrared imaging and dealing with objects embedded in turbid media.

Over the past decade, single-pixel imaging (SPI) has demonstrated its viability in scenarios where traditional imaging techniques struggle to provide images with acceptable quality in practicable times and reasonable costs. Examples of this can be found in exotic regions of the light spectrum, such as infrared^1^, terahertz^2^ or X-rays^3^. Moreover, single-pixel setups have also been proposed in challenging frameworks such as photon-counting regimes^4^, imaging in scattering media^5^,^6^, multidimensional imaging^7^,^8^,^9^,^10^,^11^, two-photon microscopy^12^, photoacoustic imaging^13^,^14^, and spatial entanglement imaging^15^.

In SPI, the resolving power of the system is shifted from the sensor to a set of microstructured spatial masks that are codified onto a programmable spatial light modulator (SLM). The masks are optically projected onto the sample and the whole intensity is collected onto a bucket (single-pixel) sensor. The photodetector provides an electrical signal, proportional to the total amount of light that leaves the object. Measurements are sequentially made by changing the spatial mask. If many different masks are used, their shapes and the electrical signal can be combined to retrieve the sample^16^.

However, SPI still has several limitations inherent to the technique. If unmodulated light arrives to the object and it is collected, the signal provided by the detector is corrupted and the recovered image gets affected. Depending on the quantity of ambient light, recovery can be unattainable. Furthermore, the use of SLMs to measure the projections of the scene onto a set of spatial masks places an upper bound to the acquisition speed of the devices. The nature of SPI enforces a reciprocal relationship between the frame rate and the image size as the time required to capture an image scales with the number of pixels in the image. As a matter of fact, SPI usually relies on the use of fast SLMs such digital micromirror devices (DMDs) to codify the projecting masks. DMDs permit highly flexible codification of binary masks at frame rates above 20 kHz. For images about 128 × 128 pixels, typical acquisition times tend to be around one frame per second^1^.

Two different approaches can be employed to overcome the above issue. On the one hand, given some reasonable assumptions about the sparsity of the signal, compressive sensing (CS) dramatically reduces the number of measurements needed well below the number of pixels of the sample^17^,^18^,^19^. What is remarkable here is that with the only assumption that the signal is sparse, it is possible to avoid the measurement of the full-length signal, saving measurement time. More recently, adaptive sensing has been introduced as a way to circumvent the computational complexity in convex optimization or greedy algorithms used in CS^1^,^20^,^21^. The idea is to make some preliminary low-resolution measurements, and take advantage of the obtained information to avoid irrelevant acquisitions in further measurements. In this way, high resolution images can be obtained in a fast and computationally efficient way.

When dealing with light coming from the surroundings, measuring directly in the Fourier space and computationally filtering the frequencies of the ambient light has been proven to increase the quality of the images even when the ambient light has more power than the signal itself 22. Moreover, the use of complementary (or differential) measurements, which has been reported in several works^1^,^9^,^23^,^24^,^25^, also increases the signal-to-noise ratio of the system, helping the devices in this type of unfavorable environmental conditions.

Here we present a novel approach, using complementary measurements and a single balanced detector. By using balanced detection, we improve the frame rate of the complementary measurement architectures by a factor of two, and we make use of all the photons arriving from the object. Furthermore, the use of a balanced detector provides environmental light immunity to the method. The key to success is the use of both reflecting arms provided by the DMD simultaneously, via the two sensors in the balanced detector. In addition to this, balanced detection also improves the signal digitization process. When we consider a single detector the detected light will be, on average, half the energy of the object with oscillations caused by the overlapping of each pattern with the object intensity distribution. The electrical signal as a function of time can be visualized as a DC term, which depends on the total energy of the object, and the AC term caused by the varying overlap of the patterns with the object. There are situations where using this approach causes loss of information because the sensitivity and dynamic range of both detectors and digitization systems are limited^6^. When using balanced detection, the DC term can be eliminated, and the full dynamic range of the detection system can be used to measure the small oscillations that are of interest to recover the image. This is another example of SPI taking advantage of the use of dedicated sensors that are difficult to implement in an array^8^,^9^,^26^.

## Results

### Experimental setup

**Figure 1:**
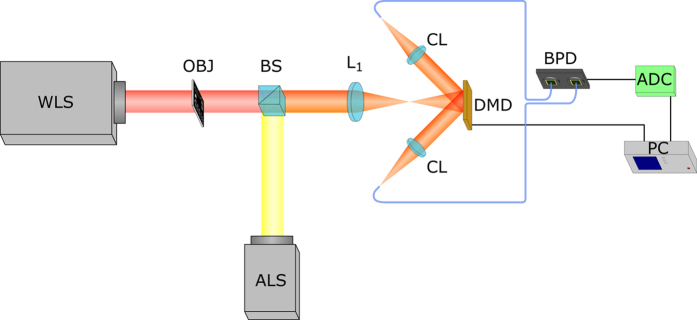
Single-pixel balanced detection camera. The object illuminated with incoherent light is imaged onto the surface of the DMD via a lens. Light reflected in both directions by the DMD is gathered by two collecting lenses and focused onto the entrance of two optical fibers, which are connected to the balanced photodetector. The signal is digitized and stored in the computer, which controls all the process. An external light source is used to introduce ambient light to the system in a controlled way with the aid of a beam splitter. ALS: ambient light source (halogen lamp), WLS: white-light source, OBJ: object, BS: beam splitter, *L*_1_: lens, CL: collecting lens, DMD: digital micromirror device, BPD: balanced photodetector, ADC: analog-to-digital converter, PC: computer.

Our single-pixel camera can be seen in Fig. 1. The system resembles a conventional camera but the object is imaged over an array of micromirrors as opposed to the traditional array of photosensors. The object is illuminated by a white-light lamp (Thorlabs HPLS200). Then, the image is created onto the DMD surface (DLP Discovery 4100 V–7000 from ViALUX). A DMD consists of an array of electronically controlled micromirrors that can rotate about a hinge. The angular position of a specific micromirror admits two possible states (+12° and −12° respect to a common direction which will be referred ON and OFF respectively). In this way, the light can be reflected in two different directions depending on the signal applied to the mirror. If one looks at the DMD from one reflection direction, only the mirrors in the ON state will reflect light, and the mirrors in the OFF state will appear dark. In the other reflection direction there will be a complementary illumination pattern, as previous OFF mirrors will reflect light, whereas previous ON mirrors will not. A sequence of sampling patterns is codified onto the DMD and the irradiance striking the large-area single-pixel photodetector is stored. In one reflection direction, we have the superposition of one pattern with the object. In the other direction, we have the superposition of the complementary pattern and the object. Light is collected by two identical collimating lenses (Thorlabs F810SMA-635), and enters two optical fibers (Thorlabs FT200EMT). The output of the balanced photodetector (Thorlabs PDB210A/M) corresponds to the subtraction of the two signals in the analog domain, amplified before the analog-to-digital conversion made by our digital acquisition system (National Instruments NI6251). In order to introduce ambient light in a controlled way, we used a commercial halogen lamp. Custom software written in LabVIEW controls both the generation of the patterns in the DMD and the digitization process of the analog-to-digital converter (ADC).

Concerning the measurement patterns, various matrices can be employed. For instance, raster-scan style masks stem from the well-known raster-scan technique in which spatial pixels are measured sequentially. Random matrices can also be used in which each mask has a random distribution of binary values. On the other hand, Hadamard matrices provide a convenient codification framework because binary non-negative elements can easily be displayed onto the DMD (see Supplementary Information). Structured matrices avoid the need to store entire matrices as the entries can be computed on the fly and permit the use of recovery algorithms with a lower computational penalty than random matrices. Note that operation at video rates is of paramount significance in potential applications of SPI like optical microscopy.

One important feature of the setup is the light collecting system. In our setup, light reflected by the DMD is collected using two identical optical fiber collimating systems. Those systems are coupled to two identical optical fibers. As both the optical fibers and the collimating systems have low numerical aperture (0.39 and 0.25 NA, respectively), light coming from directions other than the object is partially removed from the system before arriving to the detector. Even though this is a good feature and improves the quality of the presented images, it is not possible to reduce the NA of the system arbitrarily. A lower NA will decrease the field of view of the system, and also the total quantity of light at the detector, decreasing the overall quality of the reconstructions.

The other key element of our setup is the balanced detector. The use of balanced detection in optical experiments dates from the late 60’s^27^. The simplest way of doing balanced detection consists of connecting two photoreceptors in a way that their photocurrents cancel. If one is able to equalize the optical power arriving at each sensor, the effective photocurrent generated by the pair will be zero. Changes in the optical signal between both photoreceptors will unbalance the detector, generating a signal at the detector output. In this way, even if the optical power arriving at the detector is high, the output signal will only depend on the intensity of the fluctuations. This is of paramount usefulness when the information that one wants to measure relies in the light fluctuations, and not in the absolute value of the signals. Several optical techniques that require high signal-to-noise ratio have been using the principles of balanced detection for decades now. Examples range from microstructure metrology^28^, light scattering microscopy^29^, high sensitive measurements using noisy sources^30^, and what may be the most well-known optical technique using balanced detection, optical coherence tomography^31^.

In our technique, the information about the object is encoded in the difference between two signals (see Methods). In one reflection direction of the DMD, we have the result of overlapping the object and one set of patterns. On the other reflection arm, we have the result of overlapping the object and the complementary set of patterns (switching white parts by black parts and vice versa). The difference between these two signals provides the mathematical projection of the object in our basis of functions: measuring this difference using balanced detection is straightforward. Using this idea, not only we capture all the photons reflected by the object and measure both signals at the same time (reducing acquisition time), but we also use the full dynamic range of our ADC. In a traditional SPI scheme (whether using complementary illumination or not), electrical signals provided by detectors will always be positive. ADC systems use a determined number of bits in order to digitize the signal. Once the voltage range is set, the number of quantization bits determines the possible digitized voltage levels and their precision. As voltage signals have both positive and negative values, using any ADC to measure an always positive electrical signal implies losing half the number of quantization values. However, in our setup we work directly with the subtraction of two signals, which will have both positive and negative values depending on the patterns generated in the DMD. The use of both reflection arms of the DMD improves the signal strength because we utilize the full flux of photons coming from the scene, in contrast with the classical single-pixel approach, which loses, on average, half of the photons.

**Figure 2:**
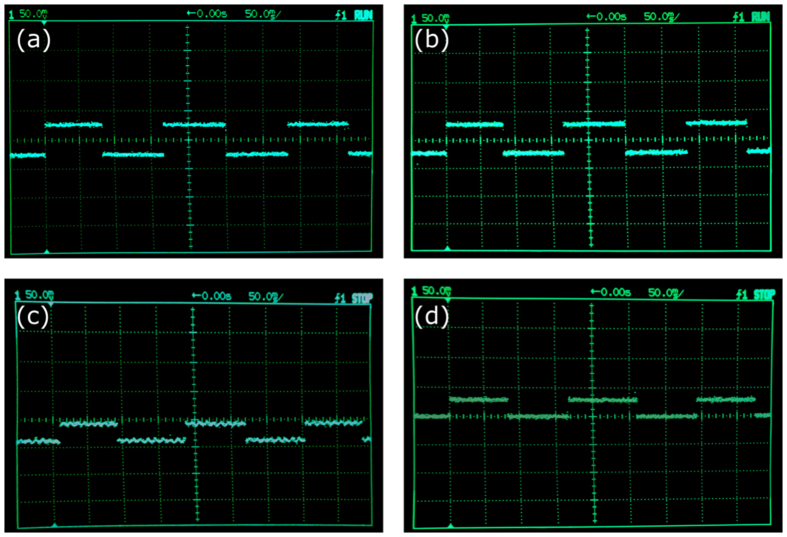
Electrical signals in presence of ambient light. Four snapshots from the oscilloscope with different illumination and sensing conditions. In order to acquire the signals, two different Walsh-Hadamard patterns were generated periodically onto the DMD surface, thus giving a low frequency square wave. The first row of pictures corresponds to balanced detection with the halogen lamp ON (**a**) and OFF (**b**). It is clear that no fast variations appear in any case. The second row of pictures corresponds to sensing with only one of the detectors when the lamp is ON (**c**) or OFF (**d**). It can be seen that a ripple at a frequency of 100 Hz appears in (**c**). If one needs to measure the difference between the two intensity levels, this oscillation can corrupt the measurement, and thus decrease the quality of the recovered image.

Lastly, the adoption of balanced detection also increases the ambient light immunity of the optical system. Since both sensors of the balanced detector work at the same time, temporal fluctuations of the signal are subtracted automatically. This is very relevant in experimental conditions where light coming from unstable sources can corrupt the signal, making unfeasible the recovery of the object. This is illustrated in Fig. 2. We show several snapshots of the electrical signal when using balanced detection or single detection and switching ON or OFF an ambient halogen light source with intensity fluctuations at 100 Hz. In all four images, we see the temporal square signal generated by the DMD when switching between two different Walsh-Hadamard patterns at a low rate (around 10 Hz). When we turn on the halogen lamp, an undesirable ripple appears in the signal, with a frequency of 100 Hz. When using balanced detection, as the oscillating signal introduced by the halogen lamp is the same in both sensing arms, it does not contribute to the balanced signal. However, in the single detection case, this ripple persists. Using the corrupted signal to recover an object via SPI techniques would provide low quality images, as we will see in the following section.

### Experimental results

**Figure 3:**
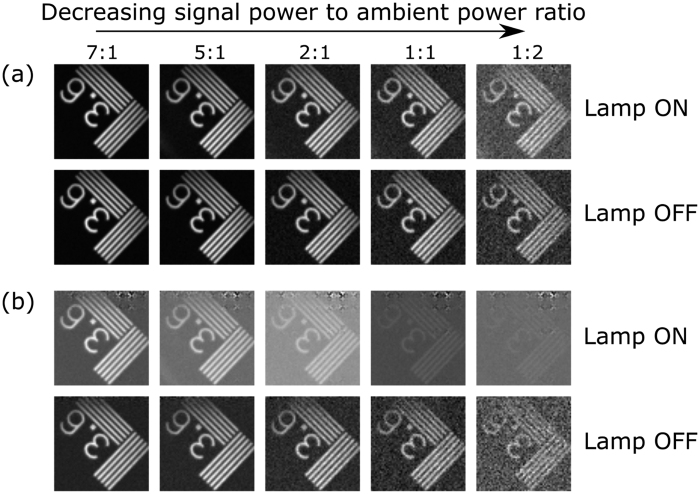
Single-pixel imaging in presence of ambient light. Series of measurements using balanced detection (**a**), and a single sensor (**b**). For each experiment, both measurements with the halogen lamp turned on and off are shown. Each column represents a different power level of the source illuminating the object, as the power of the halogen lamp is either fixed (lamp on) or zero (lamp off). In order to ease visualization, all images have been normalized between 0 and 255.

As a proof-of-concept, we used the experimental setup shown in Fig. 1 to record a set of images with different illumination conditions. The results are shown in Fig. 3. For a given fixed power of the halogen lamp, we changed the intensity of the light illuminating the sample object (a small part of an USAF resolution test chart with 3.6 lines/mm) and acquired an image both using balanced detection (Fig. 3(a)) and only with one sensor (Fig. 3(b)). In both cases, we also took images with the halogen lamp turned off. The images have a size of 64 × 64 pixels, and each pixel was arranged by grouping 4 DMD mirrors, with 13.68 *μm* width each. In order to acquire the images, the full set of 4096 (64^2^) Walsh-Hadamard patterns was generated in the DMD. To ease visualization, all the images were normalized between 0 and 255 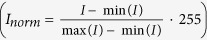.

It is clear, even when the halogen lamp is much dimmer than the object source, that the non-balanced image has much lower quality. One can see a checkerboard-like artifact at the top of the images. This is caused by the temporal oscillation of the signal at a frequency of 100 Hz. As the sampling rate of the ADC is known, one could digitally filter the 100 Hz component of the signal in the Fourier domain, go back to the time domain and recover the object suppressing this checkerboard pattern. However, this requires *a priori* knowledge of the power spectrum of the ambient and parasitic light sources. Balanced detection makes this post-processing of the electrical signal unnecessary. This is advantageous as post-processing procedures are time consuming, decreasing the total acquisition speed of the system.

Readers may observe a reduction in the quality of the images as the WLS power decreases while that of the halogen lamp remains constant. Here we point out two different effects. On the one hand, we have the effect of taking an image using a small quantity of light. On the other hand, we identify the effect of varying the power ratio between the two sources. For the first effect, we can look at both the second and fourth rows of Fig. 3. In this case, the halogen lamp is turned off and we take images decreasing the power of the lamp illuminating the object. We can see that as illumination decreases, noise starts to appear covering the image. This is due to the signal power approaching the level of the technical noise of the setup, which ultimately limits the working range of illumination powers in which the camera can acquire images. In order to work at different illumination levels or other regions of the electromagnetic spectrum, sensors can be exchanged with high sensitive photodiodes (or avalanche photodiodes) or even detectors with different spectral sensitivities (outside the visible region). The second effect depends on the ratio between the power of the two light sources. As we stated before, the halogen lamp produces a checkerboard-like pattern on top of the image when measuring using only one detector. If one compares this pattern in the third row of pictures, it is clear that decreasing the power of the lamp illuminating the object also increases the weight of the checkerboard pattern in the recovered image. So, with ambient lights much dimmer than the light coming from the object, one can recover an image which resembles the object with a small artifact coming from the ambient light. However, if the signal coming from the object decreases, artifacts will hide the object under study. This does not happen in the balanced detection case, as the sensor eliminates the fluctuating signal coming from the halogen lamp. Due to this, all the recovered images present similar quality, no matter whether the halogen lamp was turned on or off.

**Figure 4:**
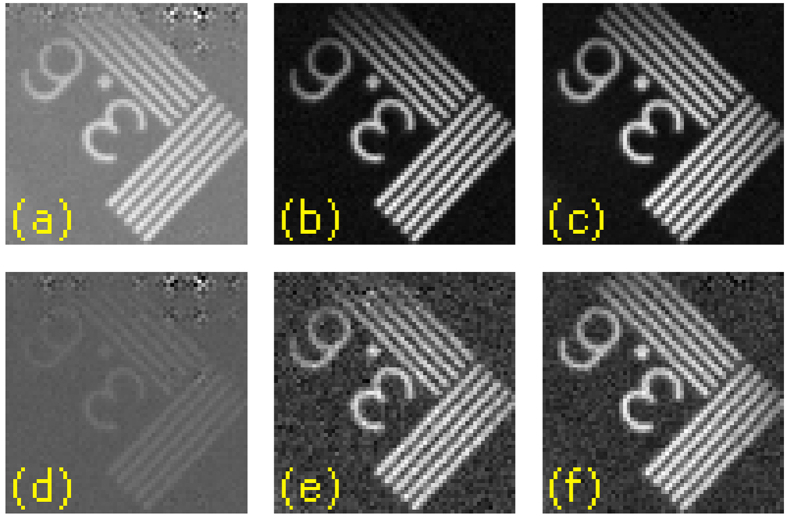
Single-pixel imaging and complementary single-pixel imaging comparison. In the first row, we show images acquired when light coming from the object has 5 times more power than the ambient light. (**a**) Single-pixel image, (**b**) sequential complementary single-pixel image, and (**c**) simultaneous complementary image. In the second row, we present the same experiment but with images acquired when the light coming from the object has the same power as the ambient light. Note that even though second and third columns have very similar quality, the simultaneous complementary image is acquired in half the time.

At this point, it is interesting to compare the results of both complementary single-pixel imaging as has been reported^1^,^9^,^20^,^23^,^32^ and our approach using balanced photodetection. Some previous work on complementary measurements worked with only one reflection arm of the DMD^1^,^9^,^20^,^32^, which entails the generation of each pattern and its complementary sequentially. In addition of doubling the acquisition time of the system, if the scene changes between a pair of complementary patterns, the coefficient associated with that pair will be corrupted. Even in the case of using both reflection arms of the DMD and two detectors^23^, measuring in a non-balanced scheme introduces both electrical and quantization errors in the process. As the complementary measurements are made at the exact same time using the balanced detector, the technique presents a natural double frame rate and temporal immunity when compared to the sequential complementary approach. Furthermore, the SNR increase stated in the previous section also improves the quality of the recovered images. We show a comparison between non-complementary, sequential, and simultaneous complementary imaging in Fig. 4.

Images shown in Fig. 4 have a size of 64 × 64 pixels. The total number of projections onto the Walsh-Hadamard basis established by the Nyquist-Shannon criterion is 4096. In order to take the single-pixel traditional image, we generate the 4096 patterns by placing white and black pixels onto the +1 s and −1 s entries of the Walsh-Hadamard matrix (shifting and rescaling approach, see Methods). In the sequential case, we generate 8192 patterns and measure the projections only by using one of the reflection arms of our setup. In the balanced setup, the first set of 4096 patterns already generates the full set of 8192 projections by using both reflection arms of the DMD. It is clear that, as stated on the published reports, the complementary approach improves the SNR of the images^23^. Both sequential and balanced approaches provide very similar results, but the balanced approach works at double frame rate and it is insensitive to temporal oscillations caused by ambient and parasitic light.

**Figure 5:**
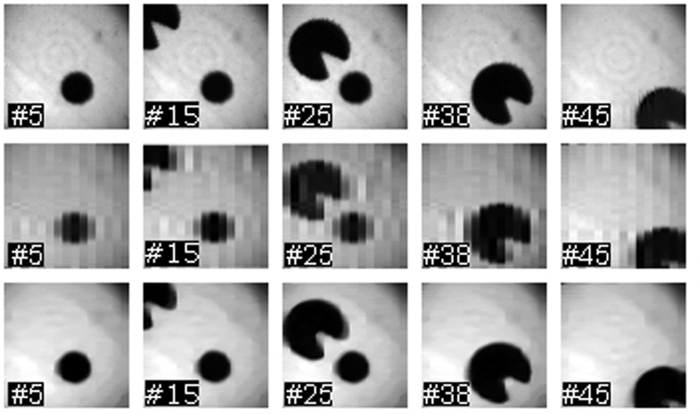
Balanced single-pixel video imaging. We show multiple frames of a live video taken with our single-pixel camera. In the top row, we show the frames acquired without using subsampling techniques. As the images consist of 64 × 64 pixels, the 4096 measurements required to take a frame were performed in roughly 200 ms. In the middle row we show frames of the same scene acquired by subsampling the scene. In this case, we used 820 patterns, which correspond to a 20% compression ratio. Each frame was acquired in 40 ms. In the lower row we show a CS reconstruction of the frames obtained before. In this case, the quality drop is almost negligible and we still can record a video at 25 Hz. The numbers shown correspond to the frame number inside the video.

Up to this point, we have remarked the improvement in quality produced by the use of balanced detection. However, there is another key feature of the technique, and it is the increased acquisition speed of the system. By using both reflection arms of the DMD in conjunction with the balanced detector, one can carry out complementary measurements at the same speed as single measurements. By doing this, we gather more information about the object at every pattern generated on the DMD, without decreasing the overall frame rate of the system. To show this benefit, we acquired a video of a moving scene with our setup (see Supplementary Information). We show some of the frames in Fig. 5. In this case, the scene consists of a moving Pac-Man eating a pellet. Every frame of the video is a 64 × 64 pixel image. We show both SPI and CS acquisitions. The total number of patterns needed to recover a frame is 4096 (64^2^), as stated by the Nyquist-Shannon criterion. As our DMD is working at a frequency of 20 kHz, roughly 200 ms are needed to acquire a frame. By doing this, we can achieve loss-less frame rates of 5 Hz.

If more speed is needed, there are several approaches that can boost the frame rate at expense of some quality loss. Here we show two more acquisitions by using undersampling and a CS approach. In order to capture a frame, a subset of the total Walsh-Hadamard basis with low spatial frequencies was generated on the DMD. In this case, we used 20% of the total number of patterns. By doing this, we can achieve frame rates of 25 Hz (second row of Fig. 5). It can be seen that even though the quality decreases, the objects can still be clearly identified. The benefit of this approach is that no post-processing is needed, so the image can be displayed in real time. This is a good approach for using the camera in a microscopy-like setup, where the user wants to search for zones of interest in a sample in a fast way. It is possible to perform real-time visualization at 25 Hz, and once the region of interest is in the field of view of the system, perform a loss-less acquisition at 5 Hz. There also exist several ways of getting high frame rates without losing so much quality in the process. In the third row of Fig. 5, we show several frames acquired with the same subset of patterns (a 20% of the total set) but recovered by means of CS. Even though the acquisition speed is 25 Hz, the post-processing time needed to use the CS algorithm hinders the live visualization of the scene.

**Figure 6:**
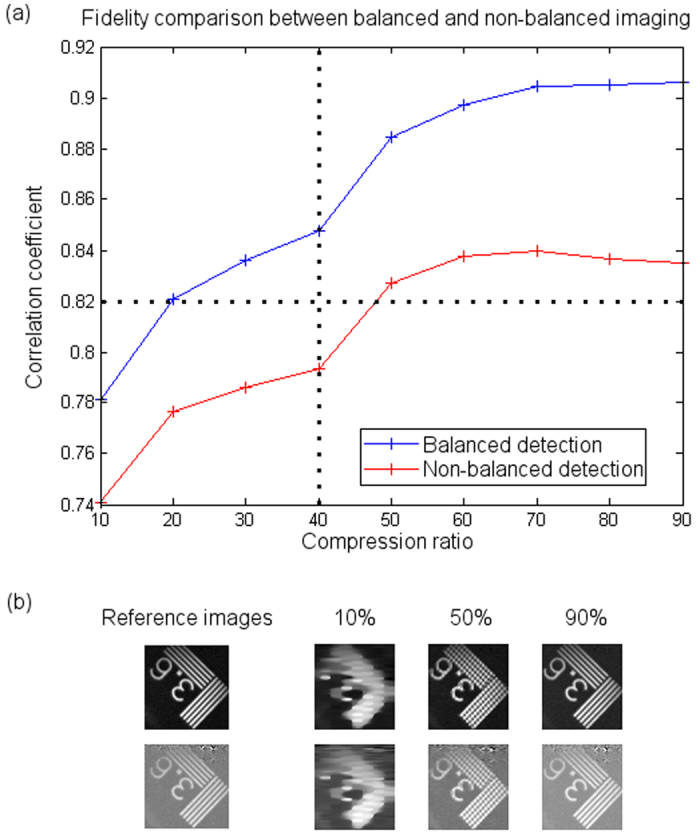
Balanced detection compressive imaging. (**a**) comparison between the correlation coefficient of several images with a reference lossless image in both balanced and non-balanced approaches. Vertical and horizontal dotted lines are included to ease the comparison between both approaches. For the same compression ratio, the balanced approach provides higher fidelity. In order to get the same quality, the non-balanced approach needs to perform more acquisitions. In (**b**) we show both reference images for balanced and non-balanced imaging, and different undersampled images (with 10%, 50% and 90% compression ratios).

Lastly, in Fig. 6 we show a comparison between the quality obtained by CS techniques whether performing balanced or non-balanced detection. In order to quantify the faithfulness of the undersampled images, we use the correlation coefficient of each undersampled acquisition with the lossless image (see Methods). The comparison between balanced and non-balanced imaging can be seen in Fig. 6(a). Even though both curves have the same behavior, it is clear that the balanced scheme improves the fidelity of the recovered images. For example, if one looks at the vertical dotted line in the graph, for the same compression ratio, the non-balanced acquisition presents lower correlation coefficients. Watching to the horizontal dotted line, it can be seen that in order to acquire the same correlation coefficient, the non-balanced approach needs to perform a higher number of measurements. In Fig. 6(b) we show both the balanced and non-balanced reference images and three of the undersampled images recovered by CS.

Here we have shown a CS approach, but several adaptive techniques can be used, with the benefits of both increased frame rate and also real time visualization, as they need negligible post-processing times^1^,^21^. As balanced detection works on the technical side of the camera (i.e. in the experimental setup), those adaptive approaches can directly benefit from this approach, doubling their frame rates and increasing the SNR of their measurements. In the Supplementary Information, we show the video comparison of the Pac-Man scene in the three cases explained here. With reduced resolutions of 32 × 32 pixels, we could achieve frame rates of 20 Hz without undersampling. With measurement ratios about 20%, which are common in the literature, we could perform live video at frame rates of 100 Hz, enabling the capture of low-resolution live biological processes.

## Discussion

We have implemented balanced photodetection in a single-pixel architecture for the first time. In conjunction with proper collecting optics and complementary single-pixel imaging techniques, we have studied the benefits of the setup when imaging in presence of ambient light. Similar techniques based on single-pixel detection have tackled the same problem with good results^22^. However, the presented method does not require neither post-processing of the signal nor any *a priori* knowledge of the nature of the ambient light to work. We anticipate that this immunity to parasitic light will be of paramount interest in low-light-level scenarios or when an object is hidden inside a scattering medium, where single-pixel approaches have already started to be used^4^,^5^,^6^. The use of complementary measurements allows us to increase the signal-to-noise ratio of our measurements. Although similar proposals have been reported previously^1^,^9^,^20^,^23^,^32^,^33^, the implementation shown here increases the frame rate of the devices by a factor of two. We have been able to obtain 64 × 64 images at a frame rate of 5 Hz without using advanced undersampling techniques. As the proposed technique only requires technical adaptation of the experimental setups, novel digital approaches, ranging from CS to adaptive techniques^1^,^20^,^21^, can benefit from both the SNR and speed boost presented here. Lastly, this kind of setup can be easily adapted to different spectral zones. For instance, inexpensive single-pixel detectors are already available in the infrared region. Therefore, we expect that SPI techniques will be able to provide full real-time imaging feedback for both medicine and industrial applications in the near future.

## Methods

### Single-pixel and complementary single-pixel imaging

Consider an object represented by a *N*-pixel image. This image can be arranged in a *N* × 1 column vector 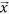. We can express this vector in an orthonormal basis of functions 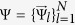. We can represent this in matrix form as 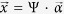, where Ψ is a *N* × *N* matrix that has the vectors 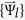 as columns and 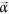 is the *N* × 1 vector with the expansion coefficients of 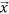 in our chosen basis. To acquire an image, SPI techniques carry out those *N* projections using an SLM, and recover the image of the object by multiplexing the acquired information. In our experiments, we work with the Walsh-Hadamard basis. The basis is conformed by orthogonal discrete square waves, with values either +1 or −1. As our SLM is binary and works by reflection, positive and negative reflection values cannot be readily implemented. To address this, two approaches can be used. The first one consists of shifting and rescaling the Walsh-Hadamard matrices so all their entries consist of either 1 or 0 values^7^. The second one, which we call complementary sensing, consists of generating a pair of matrices, related to the Walsh-Hadamard matrix by a subtraction operation. It has been reported that the complementary scheme improves the signal-to-noise ratio of the measurements and its better suited to use CS strategies^23^.

The process goes as follows. We have a Walsh-Hadamard matrix, *H*, whose entries are either 1 or −1. We create the complementary pair *H*^±^ = (*E* ± *H*)/2, where *E* represents a matrix with all entries equal to 1. By doing this, we have one matrix *H*^+^ where the original 1 entries preserve their value, but the −1 entries become zero valued. In the other matrix *H*^−^, unity-valued entries will become zero valued, and −1 entries will have a value of 1. It is trivial to prove that all three matrices are related by *H* = *H*^+^ − *H*^−^. As the SPI measuring process is linear, one can acquire the projection of the object under the Walsh-Hadamard basis by measuring the projections under *H*^+^ and *H*^−^ and calculating the difference between them.

This procedure reduces the noise introduced by light coming from outside the scene under study. Consider an object, *X*, expressed as a square matrix. Under homogeneous illumination (*P*_0_), and considering the parasite light as homogeneous additive noise (*N*) added after object illumination, the light distribution in the DMD plane will be given by *F* = *X* · *P*_0_ + *N* · *E*. Here the products are done element-wise. The measured intensities will be *I*^±^ = *P*_0_ · *X* · *H*^±^ + *N* · *E* · *H*^±^. Given the nature of the Hadamard matrices, *E* · *H* = 0, and by using this property it is easy to prove that the subtraction of the two signals is given by 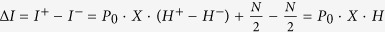.

### Compressive Sensing

CS provides a method to acquire a picture with *M* < *N* measurements, with quality determined by the measurement ratio 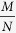. By choosing a measurement basis 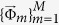, we can express the measurement process as


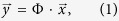


where 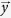 is a *M* × 1 vector containing the measured projections and Φ is a *M* × *N* matrix called sensing matrix. As the transformation from 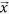 to 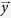 entails a dimension reduction, there is loss of information in the process. Given that *M* < *N*, there exist infinite 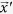 that satisfy 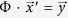. CS theory demonstrates that it is possible to recover an approximation to 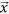
17.

To recover the image 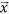 one needs to solve the underdetermined matrix relation obtained after the measurement process. There are several methods to solve this problem, such as basis pursuit or *Dantzig Selector*^34^. When working with images, it is also possible to use a model based on the gradient sparsity. Once the discrete gradient of the image is estimated, it is possible to minimize the total variation (TV), which works as a merit function of the gradient, and recover the approximated object. In our experiments, we use the Min-TV with equality constraints algorithm^35^, that solves the following problem


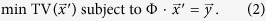


### Image comparison by correlation coefficient

In order to compare image acquisitions, we have used the correlation coefficient of the undersampled images with a reference image. This coefficient ranges from zero to one, depending on the resemblance of both images. Our reference image is always the image acquired without undersampling (i.e. measuring at the Nyquist-Shannon criterion). The correlation coefficient is calculated with the *corr2* Matlab function


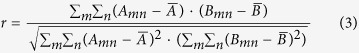


where A and B are the image matrices with indexes m and n. 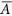, 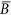 represent the mean of the elements in A and B.

## Additional Information

**How to cite this article**: Soldevila, F. *et al.* Computational imaging with a balanced detector. *Sci. Rep.* **6**, 29181; doi: 10.1038/srep29181 (2016).

## Supplementary Material

Supplementary Video

Supplementary Information
