# Supplementary material for: Computational imaging with a balanced detector
Source: Sci Rep. 2016 Jun 29;6:29181. doi: 10.1038/srep29181 (PMC4926225; doi:10.1038/srep29181)
Supplement: Supplementary Information [file srep29181-s2.pdf]

Computational imaging with a balanced detector: supplementary material

F.Soldevila[1,*], P.Clemente[2], E. Tajahuerce[1], N. Uribe-Patarroyo[3], P. Andrés[4], and J. Lancis[1]

[1]GROC·UJI, Institute of New Imaging Technologies (INIT), Universitat Jaume I, E12071 Castelló, Spain.
[2]Servei Central d’Instrumentació Científica (SCIC), Universitat Jaume I, E12071 Castelló, Spain.
[3]Wellman Center for Photomedicine, Harvard Medical School and Massachusetts General Hospital, 40 Blossom Street, Boston, Massachusetts 02114, USA.
[4]Facultat de Física, Universitat de València, E46100 Burjassot, Spain.
*fsoldevi@uji.es

Supplementary video 1

In this video we show the acquisition of the same live scene with different sampling ratios (different acquisition times for each frame). The resolution of the scene is 64x64 pixels, so the total number of measurements stablished by the Nyquist-Shannon criterion is 4096. In the first column, the sampling ratio is 100%. In the second column, only a 20% of the sensing masks are projected, thus reducing the total acquisition time of each frame by a factor 5. In the third column, compressive sensing is used in a post-processing procedure to increase the quality of each frame.
